# Inhibition of AcrAB-TolC enhances antimicrobial activity of phytochemicals in *Pectobacterium brasiliense*

**DOI:** 10.3389/fpls.2023.1161702

**Published:** 2023-05-09

**Authors:** Manoj Pun, Netaly Khazanov, Ortal Galsurker, Zohar Kerem, Hanoch Senderowitz, Iris Yedidia

**Affiliations:** ^1^ The Institute of Plant Sciences, Volcani Center, Agricultural Research Organization (ARO), Rishon Lezion, Israel; ^2^ The Robert H. Smith Faculty of Agriculture, Food and Environment, The Hebrew University of Jerusalem, Rehovot, Israel; ^3^ Department of Chemistry, Bar-Ilan University, Ramat Gan, Israel

**Keywords:** AcrAB-TolC, efflux pumps, inhibitors, naringenin, *Pectobacterium brasiliense*, phloretin, virulence, PAβN

## Abstract

**Introduction:**

The eons-long co-evolvement of plants and bacteria led to a plethora of interactions between the two kingdoms, in which bacterial pathogenicity is counteracted by plant-derived antimicrobial defense molecules. In return, efflux pumps (EP) form part of the resistance mechanism employed by bacteria to permit their survival in this hostile chemical environment. In this work we study the effect of combinations of efflux pump inhibitors (EPIs) and plant-derived phytochemicals on bacterial activity using *Pectobacteriun brasiliense* 1692 (Pb1692) as a model system.

**Methods:**

We measured the minimal inhibitory concentration (MIC) of two phytochemicals, phloretin (Pht) and naringenin (Nar), and of one common antibiotic ciprofloxacin (Cip), either alone or in combinations with two known inhibitors of the AcrB EP of *Escherichia coli*, a close homolog of the AcrAB-TolC EP of Pb1692. In addition, we also measured the expression of genes encoding for the EP, under similar conditions.

**Results:**

Using the FICI equation, we observed synergism between the EPIs and the phytochemicals, but not between the EPIs and the antibiotic, suggesting that EP inhibition potentiated the antimicrobial activity of the plant derived compounds, but not of Cip. Docking simulations were successfully used to rationalize these experimental results.

**Discussion:**

Our findings suggest that AcrAB-TolC plays an important role in survival and fitness of Pb1692 in the plant environment and that its inhibition is a viable strategy for controlling bacterial pathogenicity.

## 1 Introduction

Plants and bacteria have coevolved for over 480 million years producing a plethora of interactions between the kingdoms. To protect against pathogenic bacteria, plants produce a large array of secondary metabolites, of which phenolic compounds have been widely reported as antimicrobial agents (Pasqua et al., 2019). In response, phytopathogenic bacteria have evolved systems to deal with this rich chemical environment, including plasma membrane efflux pumps (EP) that remove toxic compounds from the cell, minimizing the pressure and facilitating survival and colonization of the host (Du et al., 2018; Pasqua et al., 2019). Large numbers of diverse and structurally unrelated compounds are excreted by EP, including disinfectants, dyes, organic solvents, detergents, bile acids, hormones, heavy metals, and a variety of defense active phytochemicals (Piddock, 2006; Alibert et al., 2017).

EP are commonly classified into five families based on their number of transmembrane spanning regions, energy sources and substrates. Of these five families, the resistance-nodulation-division (RND) superfamily is found only in Gram-negative bacteria. RND transporters are composed of a tripartite complex: an inner membrane protein, periplasmic membrane fusion protein (MFP) and an outer membrane protein (OMF). This arrangement facilitates efflux across both the inner and outer membrane, thereby conferring broad spectrum resistance to the cell (Wiggins, 2004; Zhao et al., 2021). These transporters are usually chromosomally encoded and are expressed constitutively in wild-type cells (Lomovskaya and Watkins, 2001; Wiggins, 2004; Elnasser et al., 2021).

Of the multiple RND transporters, the tripartite drug efflux complexes AcrAB-TolC and MexAB-OprM from *Escherichia coli* and *Pseudomonas aeruginosa* respectively, have been extensively studied (Li et al., 2015; Venter et al., 2015; Blanco et al., 2016; Cacciotto et al., 2018; Zhao et al., 2021). AcrAB-TolC is composed of three essential components: Homotrimeric protein AcrB which is embedded in the inner membrane and pumps out diverse substances using a proton gradient as an energy source, TolC a homotrimeric membrane protein with long cylindrical-like structure that provides conduit across the outer membrane, and AcrA which is a periplasmic adaptor protein that connects AcrB and TolC (Vargiu et al., 2014; Kim et al., 2015; Wang et al., 2017).

Similar to *E. coli*, plant pathogens in the order Enterobacterales use EP to protect the cell against the chemical environment. Such an example is *Pectobacterium brasiliense*, a Gram-negative plant-pathogen of the Pectobacteriaceae, a family of multiple genera that cause bacterial soft rot and blackleg diseases, leading to plant tissue maceration and finally wilting and collapse of the entire infected plant tissue (Lu et al., 2021; Oulghazi et al., 2021). *Pectobacteriun* along with *Dickeya* are distributed worldwide and ranked among the top 10 list of significant bacterial pathogens in agricultural ecosystems, based on their economic importance (Czajkowski et al., 2011; Mansfield et al., 2012; van der Wolf, 2021). Barabote et al. (2003) reported a role for *tolC* of *Erwinia chrysanthemi* (now *Dickeya chrysanthemi*) in survival and colonization of the pathogen in plant tissues, by protecting the bacterium from antimicrobial compounds produced by plants (Barabote et al., 2003). Similarly, AcrAB was shown as an essential component in conferring tolerance to apple phytoalexins in the fire blight pathogen *Erwinina amylovora.* Indeed, mutation in *acrB* significantly increased the pathogen’s sensitivity and attenuated its virulence (Burse et al., 2004). Comprehensive analysis of different RND efflux pumps in *E. chrysanthemi* revealed two Acr-like systems and two Emr-like systems (Maggiorani Valecillos et al., 2006). RND superfamily proteins have also been reported in other Gram-negative plant associated bacteria such as *Agrobacterium tumefaciens*, the nitrogen-fixing symbiont *Rhizobium etli*, *Bradyrhizobium japonicum and Burkholderia glumae* (Burse et al., 2004; Al-karablieh et al., 2009; Vargas et al., 2011). Complete genome sequence of *P. brasiliense* (Pb1692) has been published, with two annotated genes for *acrB* (GT391_RS01465 and GT391_RS15000) (Liu and Filiatrault, 2020).

Taking the on-going interactions between plants and pathogens to the next level, plants have developed a host of efflux pump inhibitors (EPIs). These were demonstrated to enhance the antibacterial potency of selected plant-derived and other compounds, to expand their spectrum of activity, to reduce the rates of resistance development and to reverse resistance (Lomovskaya and Watkins, 2001; Lomovskaya and Bostian, 2006; Opperman and Nguyen, 2015; Venter et al., 2015; Lamut et al., 2019). Following these previous reports, in this work we aimed to increase the activity of existing antibacterial compounds, including known plant defense molecules, by targeting AcrB of Pb 1692. To this end, we assessed the antibacterial activity of selected phytochemicals [phloretin (Pht), naringenin (Nar), carvacrol, salicyclic and *p*-coumaric acids] in the absence and presence of several known EPIs, namely, Phe-Arg β-naphthylamide (PAβN), 1-(1-naphthylmethyl) piperazine (NMP), berberine, and quinoline and found a synergistic activity between the EPI PAβN and the phytochemicals Pht and Nar. The experimental results were supported and explained by molecular docking simulations.

## 2 Results

In this work, we set out to determine the effect of EPIs on the antibacterial activity of plant-derived phytochemicals under the assumption that the simultaneous application of both types of compounds will synergistically enhance antibacterial activity. To test this hypothesis, we determined minimum inhibitory concentrations (MIC) of several plant-derived phytochemicals in the absence and presence of several known EPIs.

### 2.1 Efflux inhibition increased ethidium bromide (EtBr) accumulation 

We started by testing the activity of the EPIs on Pb1692 efflux inhibition. For this purpose, a common assay measuring the accumulation of the EP substrate EtBr inside the bacterial cells was used (Coldham et al., 2010). As shown previously by Coldham et al., 2010, the concentration of EtBr is expected to increase within the cells in correlation with EP inhibition. The positive control in these assays are dead cells, where the efflux is abolished by the lack of an active electrochemical gradient, in which EtBr accumulates undisturbed inside the cells (Reens et al., 2018). The control for the inhibitory effect is live Pb1692 cells, without exposure to EPIs, and thus, fully functional EPs. Therefore, we expect to have the highest fluorescent (i.e. EtBr accumulation) in the heat-killed bacteria and the lowest fluorescent in live cells where the EP is fully functional.

**Figure 1:**
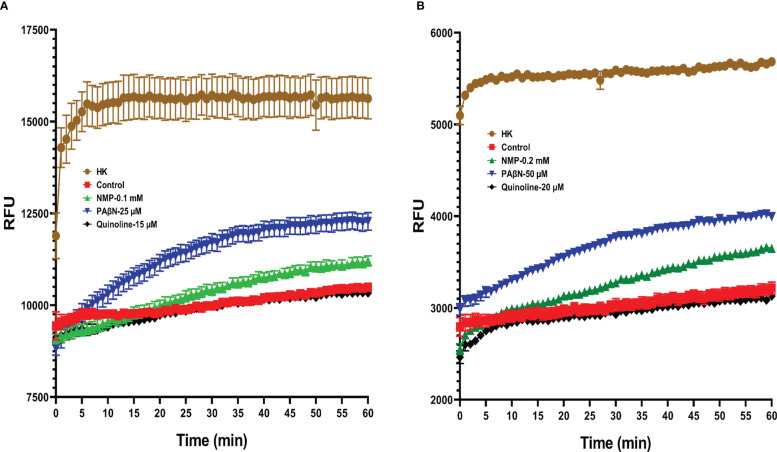
EtBr accumulation in the presence of different EPIs in *Pectobacterium brasiliense* (Pb1692), as observed by Relative Fluorescence Units (RFU). NMP, PAβN and quinoline were tested at concentrations of: **(A)** 0.1mM, 25µM and 15µM and **(B)** 0.2mM, 50µM and 20µM, respectively. All assays were performed in triplicate, and the representative results of one experiment are shown. (HK= heat killed bacteria, 1-(1-naphthylmethyl) piperazine (NMP), Phenylalanine-arginine-β-naphthylamide (PAβN) and quinoline).

EtBr accumulation (higher florescent level), revealed that PAβN was more effective in the inhibition of the efflux activity in Pb1692 than NMP or quinoline (**Figure 1**). Even at 25 μM of PAβN and 0.1 mM of NMP the efflux activity was inhibited (**Figure 1A**). In the case of quinoline, no accumulation of EtBr was observed leading to a similar pattern as the control treatment, for the tested concentrations 15 and 20 μM (**Figure 1B**). The different concentrations were selected based on the MIC results, where the highest concentration that did not show an effect on the growth of Pb1692 was selected for further investigation.

### 2.2 EPI effect on Minimum Inhibitory Concentration (MIC) of the plant derived small molecules 

**Figure 2:**
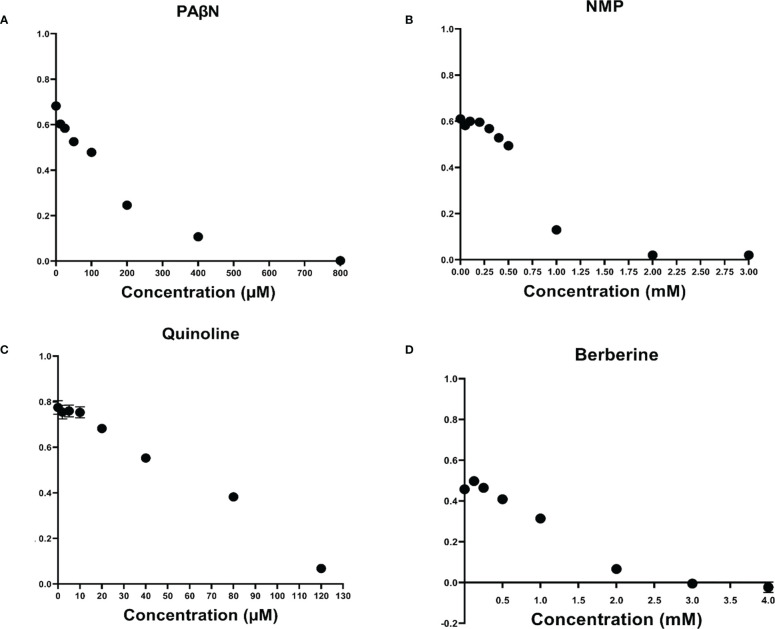
Effect of efflux pump inhibitors on the growth of *Pectobacterium brasiliense* (Pb1692). Growth (OD 600 nm) of Pb1692 at 18 h in the presence of two-fold increasing concentrations of **(A)** 1-(1-naphthylmethyl) piperazine (NMP), **(B)** Phenylalanine-arginine-β-naphthylamide (PAβN), **(C)** Quinoline, and **(D)** Berberine. Bacteria were grown in LB at 28°C, for 24 h and and growth was recorded every hour (bar = SE; n = 8). Each experiment was repeated twice. Analysis was made by GraphPad Prism 8.0.

To determine MIC of EPIs and of selected compounds, a 2-fold serial dilutions using the broth-dilution method (Clinical and Laboratory Standards Institute, 2017) was used. The results are presented in **Figure 2**, and demonstrate that from among the EPIs tested here, quinoline displayed a relatively strong inhibitory effect on Pb1692 growth, resulting in a low MIC value of 120 µM. The other EPIs studied here, PAβN, NMP and berberine, showed MIC values of 800 µM, 2 mM and 3 mM, respectively. Time dependent growth curves relative to the control treatments DMSO (1%) and dH_2_O are provided in **Supplementary Figure 1**.

**Figure 3:**
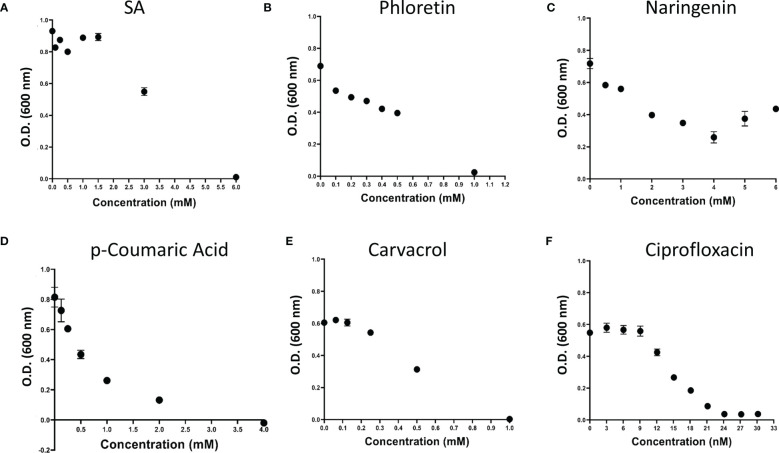
Effect of plant phenolic compounds and the antibiotic ciprofloxacin (Cip) on growth of *P. brasiliense* (Pb1692). Growth (OD 600 nm) of Pb1692 at 18 h in the presence of two-fold increasing concentration of **(A)** Salicylic acid, **(B)** Phloretin, **(C)** Naringenin, **(D)** *p*-Coumaric acid, **(E)** Carvacrol, **(F)** Ciprofloxacin. Bacteria were grown in LB at 28°C, for 24 h and growth was recorded every hour (bar = SE; n = 8). Analysis was made by GraphPad Prism 8.0.

Looking at the phenolic compounds (Nar, Pht, salicyclic and *p*-coumaric acids), a similar assay revealed only weak antimicrobial activity for Pht and carvacrol with the lowest MIC value of 1mM (see **Figure 3** and **Table 1**). The antibiotic Cip, which impairs cell division in Gram-negative bacteria was used as a positive control and presented 4 orders of magnitude higher antimicrobial activity with a MIC value of 30.2 nM (10 ng/μl). Time dependent growth curves relative to control treatments with DMSO (1%) and dH_2_O are presented in **Supplementary Figure 2.**

**Table 1:** MIC values of tested compounds and combinations.

	Compound	MIC				
		Alone	+ PAβN 50 μM	+ NMP 0.2 mM	+ Bererine 0.5 mM	+ Quinoline 20 μM
1	Phloretin	1 mM	0.2 mM	1 mM	1 mM	1 mM
2	Naringenin	>6 mM	0.5 mM	0.5 mM	2 mM	2 mM
3	Salicylic acid	6 mM	4 mM	4 mM	4 mM	4 mM
4	*p*-Coumaric acid	4 mM	2 mM	2 mM	ND	4 mM
6	Carvacrol	1 mM	500 µM	1 mM	1 mM	1 mM
7	Ciprofloxacin	30.2 nM	30.2 nM	30.2 nM	30.2 nM	30.2 nM

Next, MIC values for each of the phenolic compounds were determined in combination with each EPI (**Table 1**). The combinations that provided the most significant reduction in MIC value were those of PAβN at 50 μM with either Pht or Nar, leading to 5 and 12 fold reduction, respectively. A combination of carvacrol with PAβN resulted in reduced MIC value by 2 fold, revealing a stronger effect of the above compounds with the EPI.

### 2.3 Synergism between EPIs and the selected phytochemicals

The Checkerboard assay was used to determine whether certain combinations of EPIs with phenolic compounds exerted a synergistic effect on bacterial growth (expressed as MIC). The Fractional Inhibitory Concentration Index (FICI) was calculated from the growth inhibition results after 24 h of growth. FICI results for each combination are interpreted as follows: FICI ≤ 0.5, synergism; 0.5< FICI< 1, additive; 1 ≤ FICI< 2, indifferent; and FICI ≥ 2, antagonism. The results are presented in **Table 2** and demonstrate that combining PAβN with either Pht or Nar led to a synergistic effect, with 5 and 12 fold reduction in their MIC value respectively. The other combinations tested with PAβN led to an additive effect only. NMP also led to a synergistic effect with Nar, whereas berberine and quinoline, presented an additive effect with some of the compounds. No synergistic effect was observed neither with PAβN, nor with NMP in combination with Cip, although it is a known substrate of the EP in *E. coli*. This was reflected in a calculated FICI value of 0.75 or 0.93 respectively, supporting an additive effect.

**Table 2:** FICI values of the studied EPIs-phenolic compounds combinations.

No	Compound	FICI value			
		PAβN	NMP	Berberine	Quinoline
1	Phloretin	0.33-0.26	0.75	1.1	>0.67
2	Naringenin	≤0.16	0.436	0.626	>0.67
3	Carvacrol	0.52	≥0.6	0.54	1.16
4	Salicyclic Acid	0.682	0.72	0.87	0.83
5	*p*-Coumaric Acid	0.52	0.55	ND	0.67
6	Ciprofloxacin	0.75	0.93	1.16	1.05

### 2.4 Differential expression of *acrAB-tolC * gene components in the presence of EPIs

To explore the effect of the compounds at sub-MIC concentrations on gene expression patterns of the components of *acrAB-tolC* genes, a qPCR analysis was performed. Primers for *acrA, acrB* and *tolC* in Pb1692 were designed (**Supplementary Table 1**). The expression of the tripartite genes was evaluated at two time points (15 min, and 2 h). These were selected based on our preliminary results as well as previous findings in *E. coli* (Kobayashi et al., 2006). Based on the FICI results, two substrates of AcrAB-TolC, Pht and Nar and one antibiotic Cip, were evaluated in this assay with and without the presence of the EPIs, PAβN and NMP.

**Figure 4:**
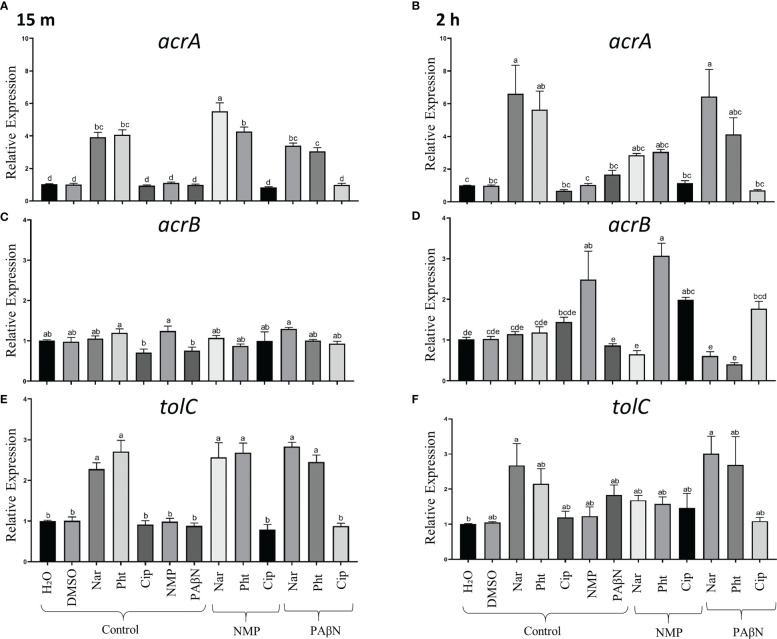
Effects of phloretin (Pht), naringenin (Nar), ciprofloxacin (Cip), NMP and PAβN alone and in combinations, on transcript levels of the tripartite genes associated with the efflux pump AcrAB-TolC in *Pectobacterium brasiliense* (Pb1692). The expression levels of *acrA*, *acrB*, and *tolC* were determined by qPCR of bacterial cultures grown in LB, 28°C at 15 min (first measurement) and 2 h, in the presence of the tested compounds or their combinations. **(A, B)** Relative expression of *acrA* at 15 min and 2 h post exposure to the compounds, respectively. **(C, D)** Relative expression of *acrB* as above, and **(E, F)** Relative expression of *tolC*, as above. Control samples were treated with dH_2_0 or 0.3% DMSO. Bars represent means + standard errors (SE) of the relative gene expression observed in three experiments, each with nine replicates per treatment. The experiments were repeated three times. One-way ANOVA with *post hoc* Tukey-Kramer HSD tests was used to analyze differences. Bars that are not labelled with the same letter are significantly different from each other (*P*< 0.05; bar = mean + SE; *n* = 9).

The results are presented in **Figure 4** and demonstrate that the expression of the efflux pump genes *acrA* and *TolC*, increased significantly at an early phase, following the application of either Pht or Nar (at 15 min and 2 h) alone, or in combination with the EPIs (NMP and PAβN) (**Figures 4A, E**, alone; B, F combined). Relative to the control treatment, PAβN treatment induced higher transcripts levels of *acrA* and *TolC* genes after 2 h (**Figures 4B, F**), and had no effect on *acrB* expression level (**Figures 4C, D**). The expression of *acrB* was increased after 2 h following the application of the antibiotic Cip and the EPI NMP (2.5 fold) relative to the application of dH_2_O. Unlike PAβN, the application of NMP at all time points, did not induce upregulation of *acrA* and *TolC* (**Figures 4A, B, E, F**). The *acrB* gene was upregulated when Cip was applied together with NMP or PAβN peaking at 2 h (**Figures 4C, D**). The combinations of the EPIs with Pht or Nar provided inconsistent results, with upregulation of the expression at 2 h only for the combination of NMP and Pht (**Figure 4D**).

### 2.5 Plant phenolic compounds in combination with EPIs, effectively reduce Pb1692 infection

**Figure 5:**
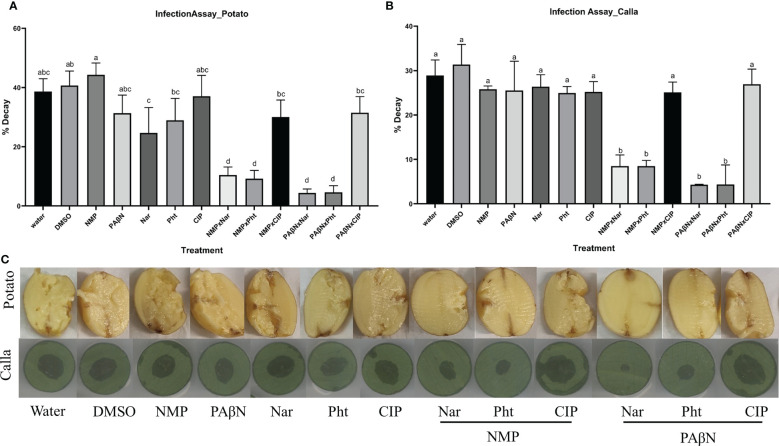
Effects of efflux pump inhibitors alone and in combinations with the plant derived compounds phloretin and naringenin (Pht and Nar, respectively) and the antibiotic Ciprofloxacin (Cip), on *Pectobacterium brasiliense* (Pb1692). Graphs represent infection of **(A)** potato tubers, and **(B)** calla lily leaf discs. **(C)** Photos of disease symptoms development in potato tubers (top) and calla lily leaf discs (bottom). Control treatment were dH_2_0 or 0.3% DMSO. One-way ANOVA with *post hoc* Tukey-Kramer HSD tests was used to analyze differences. Bars that are not labelled with the same letter are significantly different from each other (*P*< 0.05; bar = mean + SE; *n* = 40 for calla, 12 for potato).

The effect of the natural compounds and their combination with the selected EPIs (PAβN and NMP) was tested in potato tubers and in leaf discs of calla lily as hosts. Infection experiments were performed with Pb1692 according to previously described protocols (Luzzatto et al., 2007; Joshi et al., 2015). All compounds were tested alone at non-growth-inhibiting concentrations, or in combinations of each phytochemical with the tested EPI. The results revealed significant effects on disease severity. The combination of NMP at 0.2 mM and PAβN at 50 μM with either Pht or Nar led to decreased disease symptoms in both calla lily leaves and potato tubers. Cip, which is also a known substrate of AcrB, did not exert the same inhibitory effect on disease development (**Figure 5**).

### 2.6 Computational studies

#### 2.6.1 Homology modeling

**Figure 6:**
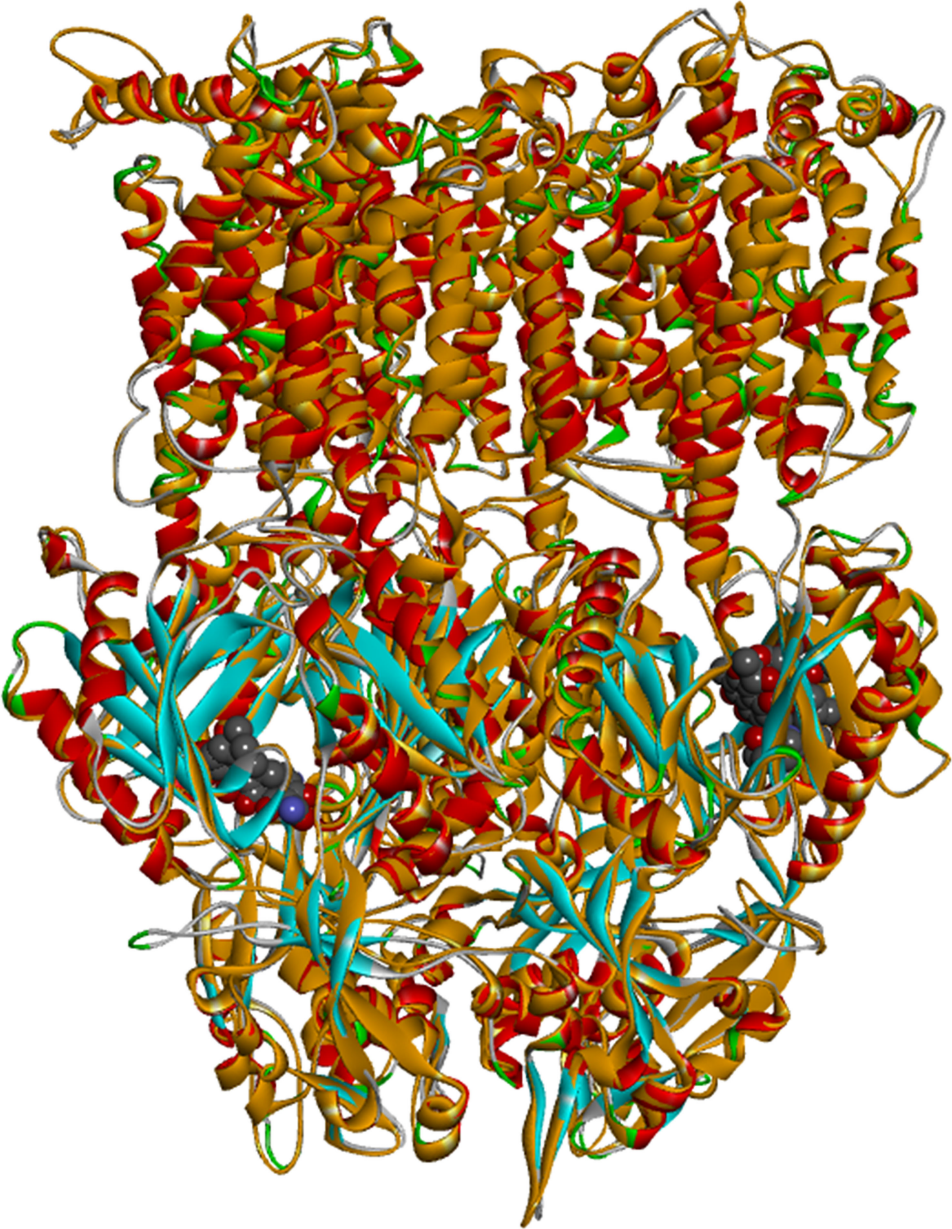
Superposition between *Escherichia coli* AcrB (PDB code: 3AOD; ribbon diagram color coded according to the secondary structure with alpha helices in red and beta sheets in blue) and the homology model of AcrB of *Pectobacterium brasiliense* Pb1692 (ribbon diagram colored in orange). The ligands (rifampicin and minocycline) are presented in CPK representation.

In order to obtain atomic-scale information on how the different EPIs occupy the binding site of AcrB as well as to rationalize the experimental findings, we resorted to molecular docking. Since the crystal structure of the efflux transporter AcrB of Pb1692 has not been solved to date, a suitable homology model was first constructed and validated based on the crystal structure of *E. coli* AcrB (PDB code: 3AOD; see **Figure 6** and the Methods section). Similar to AcrB of *E. coli*, AcrB of *P. brasiliense* is a homotrimer, with each of three monomers representing a different intermediate state along the transport process, namely, `access` (chain C) -`binding` (chain A) - `extraction` (chain B). Thus, the conformation of each of the monomers is different. The access and binding states are characterized by distinct binding sites termed the proximal and distal sites, respectively.

The resulting model was prepared for docking using Schordinger’s protein preparation wizard (see Methods section for more details) and the four EPIs considered in this work were docked into the two sites. The results are presented in **Table 3**. In agreement with the experimental data, PAβN is predicted to be the best binder into both sites with Glide scores of -5.6 (distal site) and -7.7 (proximal site) kcal/mol.

**Table 3:** Glide scores (kcal/mole) for the four EPIs considered in this work in the proximal (chain C) and distal (chain A) sites of the AcrB model.

Compound	Proximal Site	Distal Site
8-hydroxyquinoline	-5.6	-5.3
Berberine Chloride	-6.7	-4.8
1-(1-naphthylmethyl)- piperazine (NMP)	-5.3	-5.4
Phe-arg-β-naphtylamide (PAβN)	-7.7	-5.6

**Figure 7:**
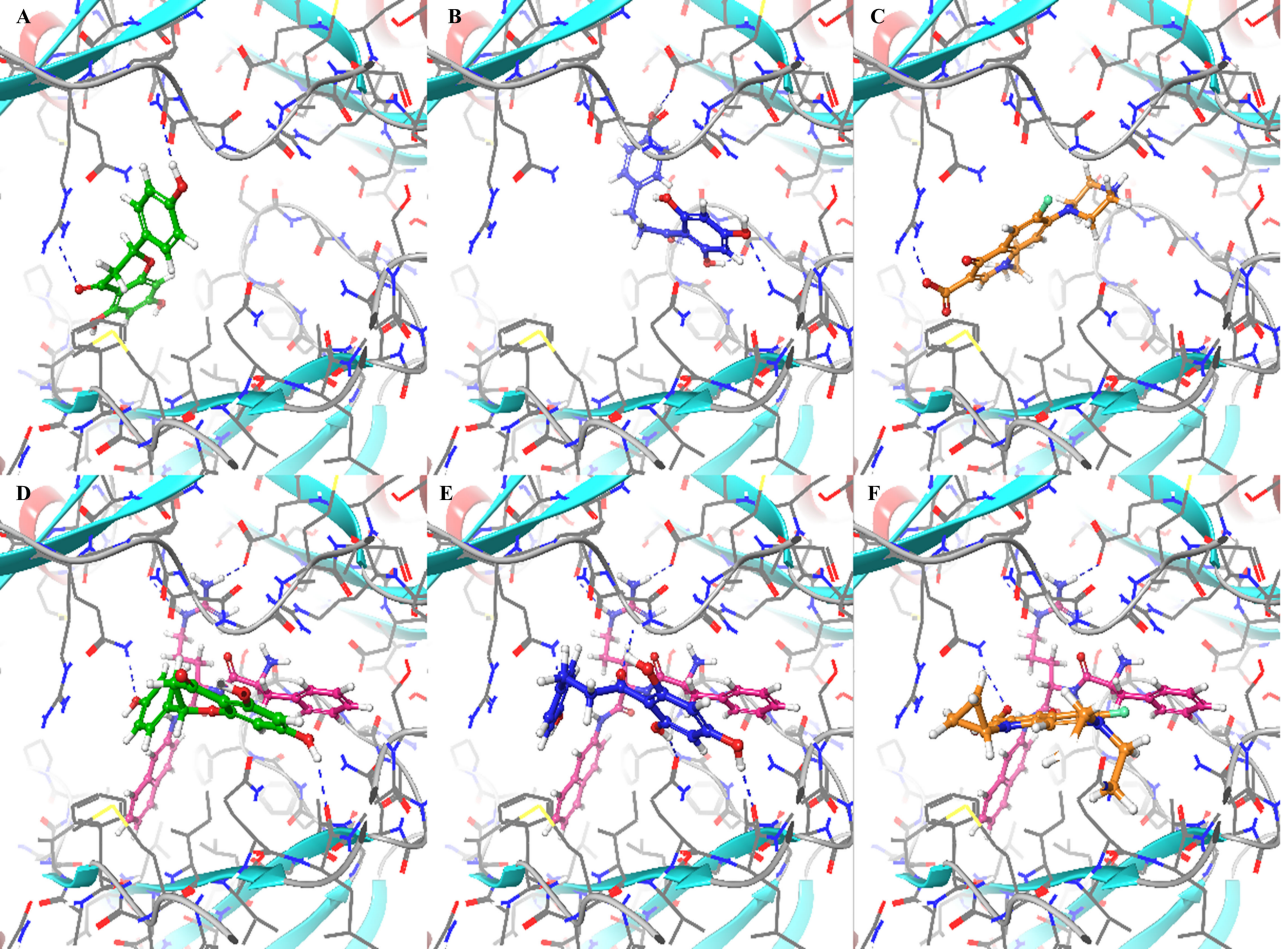
Binding modes of naringenin (Nar), phloretin (Pht), and ciprofloxacin (Cip) to the proximal binding site of the AcrB model either in the absence or the presence of PAβN: **(A)** Nar without PAβN, forms two hydrogen bonds with Arg716 and the backbone of Pro717. **(B)** Pht without PAβN, forms four hydrogen bonds with Asn622 and Glu816, and the backbone of Ser617 and Gly618. **(C)** Cip without PAβN, forms one hydrogen bond with Arg716. **(D)** Nar with PAβN, forms two hydrogen bonds with Gln827 and the backbone of Leu577. **(E)** Pht with PAβN, forms four hydrogen bonds with Gln576, Gln827, and the backbone of Leu577 and Gly719. **(F)** Cip with PAβN, forms one hydrogen bond with Gln827. The protein is presented as a ribbon diagram color coded according to the secondary structure with alpha helices in red and beta sheets in blue and the ligands are presented in a ball and stick representation. Hydrogen bonds are presented as blue dashed lines.

**Figure 8:**
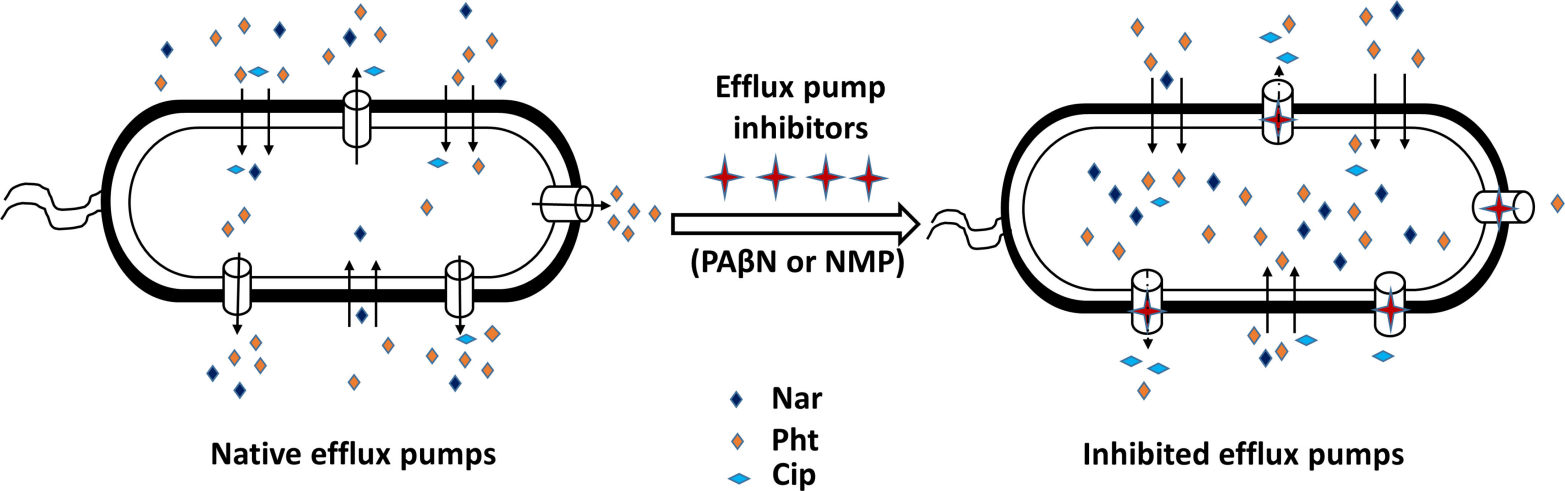
A simplified model illustrating the accumulation of phenolics from plants in Pectobaterium, following the application of synthetic efflux pump inhibitors (EPIs). The plant phenolics naringenin (Nar) and phloretin (Pht) are accumulating in the bacterium cells, while the antibiotic ciprofloxacin (Cip) is actively removed when either phenyl-arginine-β-naphthylamide (PAβN) (C) or 1-(1- naphthylmethyl)-piperazine (NMP) were applied.

The experimental results presented in this work suggest that PAβN inhibits the efflux of the two AcrB substrates, Pht and Nar, but not of the antibiotic Cip. To rationalize these observations, we docked Pht, Nar, and Cip into the proximal and distal binding sites either alone or in combination with PAβN. The results are presented in **Table 4** and **Figure 7**, and suggest stronger binding of both Nar and Pht than of Cip to the proximal site of the pump either in the absence or in the presence of PAβN. Thus, the Nar+PAβN/Pht+PAβN combinations effectively block the entrance of compounds to the access site of the flux pump, more than the Cip+PAβN combination does. The summary of these observations is provided in a simplified model (**Figure 8**), that demonstrates how in contrast to the antibiotic Cip, the plant derived compounds Nar and Pht accumulate in the bacterial cell in response to EP inhibition.

**Table 4:** Glide scores (kcal/mole) for naringenin, phloretin, and ciprofloxacin in the proximal (chain C) and distal (chain A) binding sites of the AcrB model in the presence and absence of the EPI PAβN.

Compound	Proximal Site		Distal Site	
	- PAβN	+ PAβN	- PAβN	+ PAβN
Naringenin	-6.2	-6.9	-6.3	-6.2
Phloretin	-6.7	-6.8	-5.3	-4.7
Ciprofloxacin	-5.0	-5.8	-6.0	-4.9

## 3 Discussion

Plant pathogenic bacteria inhabit a complex chemical environment, in which they are exposed to diverse antimicrobial compounds produced by both plants and competing microorganisms. In order to colonize plants and initiate disease, plant pathogens must elude these toxic effects. Efflux pumps are one of the tools shaped by evolution to counteract the hostile environment (Burse et al., 2004; Al-karablieh et al., 2009; Martinez et al., 2009; Vargas et al., 2011; Alvarez-Ortega et al., 2013). The AcrAB-TolC complex has a significant role in detoxicification of intracellular toxic metabolites, yet, other transporters also take part in protecting bacterial cells from antimicrobial compounds (Cudkowicz and Schuldiner, 2019).

In the search for novel antibacterial mechanisms, the inhibition of AcrAB-TolC and the resulting accumulation of antimicrobial phytochemicals in the pathogen’s cell is suggested and evaluated here in a plant pathogen model. To this end, the efficacy of four known EPIs, i.e. berberine, NMP, PAβN and quinoline, was evaluated in Pb1692 cells by monitoring the decrease of MIC values of several concomitantly applied antimicrobial phytochemicals. This led to the identification of PAβN and NMP as the best EPIs. Interestingly, these same compounds also exhibited a synergistic effect upon their application together with two plant derived phytochemicals, Pht and Nar (**Table 2**). The latter are well recognized substrates of the AcrAB-TolC machinery, and their increased efficacy may have resulted from the increase of their intracellular concentrations.

In *E. coli*, NMP was directly related to inhibition of AcrAB as demonstrated by reversed resistance to fluoroquinolones in an AcrAB overexpressor strain, and not in an AcrAB deficient mutant (Bohnert and Kern, 2005). The mode of action of NMP suggests that during pumping, NMP moves from the proximal to the distal pocket of AcrAB-TolC and straddles the G-loop instead of moving out. This interferes with normal substrate movement that may result in reversal of drug resistance (Li et al., 2015; Anes et al., 2019).

In contrast with Pht and Nar, the MIC value of the antibiotic Cip was not modified by the application of either PAβN or NMP in Pb 1692 (**Table 1**), suggesting that the inhibitors did not impair Cip efflux from the cell. This may suggest that the inhibitors block the passage of Nar or Pht through the efflux pathway to a higher degree than the passage of the antibiotic. In drug resistant *E. coli*, the pump preferentially removes PAβN, while the antibiotic accumulates in the cell until the concentration is sufficient to impair the target’s activity. This mode of action allows in most cases to sensitize the bacteria, or completely reverse resistance to a given compound (Bolla et al., 2011).

In order to elucidate a possible mechanism for the higher antimicrobial activity of some combinations rather than others (i.e. PAβN with Pht or Nar, and NMP with Nar), the potential existence of a feedback gene expression machinery in response to the application of both EPIs and of their combinations with the phytochemicals was studied. A 2.5 -fold increase of *acrB* expression was observed in Pb1692 following 2 h of exposure to 0.2 mM NMP (**Figures 4D**), while both *acrA* and *tolC* were not upregulated (**Figures 4A, B, E, F**). Application of PAβN did not upregulate the overexpression of *acrB* (**Figures 4C, D**), but upregulated *acrA* and *tolC* genes, by 3- and 2-fold respectively (**Figures 4B, F**). These results are in agreement with previous studies that have demonstrated in other bacteria different expression patterns of the three components of the EP in response to different EPIs (Grimsey et al., 2020; Kaur et al., 2021). Studying the feedback induced by non-growth-inhibiting concentrations of Pht and Nar revealed an early upregulation of the three components by both compounds at 15 min (first measurement point) post exposure. This up-regulation will contribute to the rapid excretion of these compounds from the cell and to increased bacterial resistance. The expression levels of *acrB* did not change when treated with Nar and Pht alone but the combination of NMP with Pht or Cip, and PAβN with Cip increased the expression of *acrB* (**Figure 4D**).

Surprisingly, the application of PAβN induced downregulation in the expression of the *acrB* gene (**Figures 4C, D**), relative to each compound alone, suggesting a mechanism for their accumulation in the bacterial cells and the observed lower MIC values in the presence of the EPI. The direct effect of Pht and Nar on EP gene expression and its down-regulation with the inhibitors, support the interference of the EPIs with AcrAB-TolC competence to protect the cells against the phytochemicals. In contrast, Cip, alone or in combination with NMP and PAβN had no effect on the expression levels of *acrA* and *TolC* at 15 min and at 2 h (**Figures 4A, B, E, F**), whereas *acrB* was upregulated after 2 h (**Figure 4D**). This result together with the observed unchanging MIC values may suggest that the EP was still effective in eliminating the antibiotic from the cell. Recent studies reported on the plasticity and redundancy of efflux transporters in *E. coli* (Cudkowicz and Schuldiner, 2019), suggesting that other transporters may be involved, mainly in plant pathogenic bacteria (Zeng and Charkowski, 2021). Upregulation of *acrB* in response to Cip treatment was also reported in *Salmonella enterica* (Ferrari et al., 2013), and upon resistance to antibiotics in *Enterobacter cloacae* upon exposure to cefepime (Liu et al., 2018).

The synergistic compounds combinations with FICI values of less than 0.5, i.e. Nar with either NMP or PAβN, and Pht with PAβN, induced a downregulation of *acrB* expression after 2 h (**Figure 4D**). Downregulation of *acrAB-tolC* is commonly observed with increased susceptibility to antibiotics (Li et al., 2011; Zhou et al., 2013; Dhara and Tripathi, 2020). Here, when the bacteria are more tolerant to the antimicrobial compounds, *acrB* is upregulated as with NMP, with Cip and Pht, and PAβN with Cip, which also displays higher FICI index (FICI > 0.5) (**Table 2**).

Infection assays were conducted using potato tubers and calla lily leaves as hosts of Pb1692, and disease symptoms were recorded to verify that plants may indeed be better protected by the application of EPIs and selected phytochemicals. The results have demonstrated the efficacy of the EPIs together with recognized antimicrobial phytochemicals in attenuating disease development. Both NMP and PAβN potentiated Pht or Nar activities on two hosts (**Figure 5A, B, C**), at concentrations that did not inhibit cell growth. The potentiation by the EPIs was differential, depending on the substrate, maybe suggesting why plants produce so many antimicrobial compounds in response to pathogen attacks. Here again, Cip did not provide protection and was probably efficiently eliminated from the bacterial cells. The infection results were consistent with previous studies showing that inactivation of AcrAB-TolC or its homologues resulted in reduced disease severity in plants (Barabote et al., 2003; Burse et al., 2004; Maggiorani Valecillos et al., 2006; Brown et al., 2007; Al-karablieh et al., 2009; Vargas et al., 2011). Nonetheless, we may not rule out the involvement of additional mechanisms in response to the low phytochemicals concentrations, including the inhibition of additional EP, effects on membrane permeability, and QS interference.

Finally, computational studies served to better infer how the application of EPIs may increase the efficacy of the selected phytochemicals and not of Cip. Similar studies have been recently employed by Kinana et al., 2016, to explain the behavior of PAβN and some of its analogs as substrates of the AcrB efflux pump in *E. coli* and as modulators of AcrB-induced nitrocefin efflux (Kinana et al., 2016). Yet, these studies primarily focused on the distal site whereas this work puts more emphasis on the proximal (access) site. In the absence of a crystal structure for Pb1692 AcrB, a homology model of the pump was first constructed based on the *E. coli* AcrB crystal structure. The remarkable similarity between the two structures supports the importance of these EP for bacterial fitness even under different lifestyles and environments. Next, docking of the four EPIs (berberine, NMP, PAβN and quinoline) to the homology model, suggested PAβN to be the best binder to both chains: A (proximal site) and C (distal site) of AcrB (**Table 3**). These results are in agreement with the quantitative EtBr accumulation assay (**Figure 1**). Finally, molecular docking suggested stronger binding of both Nar and Pht than of ciprofloxacin to the proximal site of the pump either in the absence or in the presence of PAβN (**Table 4**). Furthermore, the presence of PAβN slightly increases the predicted binding affinity of the two plant-derived compounds (as well as of Cip) to this site. This may suggest that the two phenolic compounds more effectively block the efflux pathway than the antibiotics in accord with their increased accumulation in the cell when applied in combination with the EPI PAβN. Interestingly, the presence of PAβN seems to reduce the affinity of the antibiotic to the distal site facilitating its removal from the cell.

Looking at **Table 4**, we do not see the same trend in the predicted binding affinities for the proximal and distal sites. While the two phytochemicals are predicted to be better binders than the antibiotic to the proximal site either in the absence or presence of PAβN, only Nar is predicted to be a better binder than Cip to the distal site. However, since the transport process requires sequential binding to the proximal, distal and extrusion site, we suggest that the trend observed for the proximal site is sufficient to explain the experimental results.

The weaker interaction between AcrB and Cip in *E. coli* (Vargiu and Nikaido, 2012) and with tetracycline in *Klebsiella pneumonia* (Jamshidi et al., 2018) compared with PAβN has been reported, resulting in efflux of antibiotics through the transporter. Binding of PAβN to the hydrophobic pocket in AcrB causes an interference with the binding of other drug substrates similar to our findings (Vargiu and Nikaido, 2012; Jamshidi et al., 2018).

In summary, inhibition of the AcrAB-TolC pump in Pb1692 by EPIs to prevent it from excreting phytochemicals from the cytoplasm to the periplasm and the external environment, may serve as a mean by which plants control bacterial pathogenicity. Thus, efflux pumps in Pb1692 and by extension, in other plant pathogenic bacteria, may serve as plausible targets for novel and sustainable anti-bacterial targets.

## 4 Materials and methods

### 4.1 Minimum inhibitory concentration

The microplate dilution assay was employed to assess the minimum inhibitory concentration (MIC) of the various compounds as described by the Clinical and Laboratory Standards Institute guidelines (Clinical and Laboratory Standards Institute). In Each well, a total of 190 μL of LB broth containing 2-fold serial dilutions of each of the tested compounds was inoculated with 10 μL of bacterial suspension taken from 1 × 10^8^ CFU of Pb1692 solution so that the final concentration in each well was 1 × 10^6^ CFU. The wells (in 96-well microtiter plates) were incubated for 24 h at 28°C, with continuous shaking at 150 rpm. The MIC was recorded as the lowest concentration of compound that was able to inhibit the visible growth of bacteria (at OD 600 nm).

### 4.2 Time-kill test/checkerboard assay

Two-fold serially diluted concentrations of one of the combination’s components were dispensed in the rows of a 96-well plate whereas two-fold serially diluted concentrations of the other component were dispensed in its columns. Each compound was diluted to different concentrations with growth effect close to control of the previously determined MIC. Briefly, each concentration of the tested EPI compound was mixed with different concentrations of the phytochemical compounds to a final volume of 190 µl in each well. The growth of Pb1692 was assessed by adding 10 μL of bacterial suspension (1x10^8^ CFU/mL) into a well containing the combination of each EPI and tested compound. Following 24 h of incubation at 28°C, the plates were recorded. Each value was a mean of four replications out of three independent experiments.

The effects of the antimicrobial combinations were defined according to the fractional inhibitory concentration index (FICI) using the following equation:


$$FICI=\;\frac{MIC\;of\;compound\;A\;in\;combination}{MIC\;of\;compound\;A\;alone}+\frac{MIC\;of\;compound\;B\;in\;combination}{MIC\;of\;compound\;B\;alone\;}$$

### 4.3 Ethidium bromide accumulation assays

Accumulation of EtBr inside the bacterial cell, was measured according to Coldham et. al., 2010, with minor modifications. Briefly, a single colony of Pb1692 was cultured overnight at 28°C, and 10µL were transferred to fresh LB and incubated for 5 h at 28°C, under continuous shaking. Bacterial cells were collected by centrifugation at 4000 g and re-suspended in 1 mL PBS. The optical density of all suspensions was adjusted to 0.4 OD 600, and aliquots (0.18 mL) were transferred to a 96-well plate (flat-bottomed, black, Greiner Bio-one, Stonehouse, UK). The efflux inhibitors were added as follows: column 1: PBS blank; column 2: heat-inactivated Pb1692 (10 min at 90°C); column 3: control strain (No efflux inhibitor); columns 4 –12: efflux pump inhibitors at different concentrations. Eight replicates of each inhibitor were analyzed in each column. The plate was transferred to a Spark multimode microplate reader (Tecan Trading AG, Männedorf, Zurich, Switzerland), incubated at 28°C and EtBr (25 µg/ml) was added (20 µL) to each well to a final concentration of 2.5 µg/ml. Fluorescence was recorded using excitation and emission filters of 360 and 590 nm, respectively, with 5 flashes/well; readings were taken for 60 cycles with a 75 s delay between cycles, and a gain multiplier of 1460. Raw fluorescence values were analyzed using Excel (Microsoft) and included mean values for each column following subtraction of appropriate control blanks. Each experiment was repeated twice.

### 4.4 Gene expression

#### 4.4.1 RNA extraction and cDNA preparation

Pb1692 cells were grown from a single colony for 16 h, at 28°C in LB under continuous shaking. Then fresh LB, with or without EPI (NMP and PAβN), antimicrobial compounds (Nar, Pht and CIP) and their combinations, was inoculated with 5x10^8^ CFU of bacterial suspension, in 15 mL LB, and incubated for 15 min and 2 h at 28°C, under continuous shaking. After15 min and 2 h, 2mL samples were taken to an Eppendorf tube for RNA extraction. To extract RNA, GENEzol™ Reagent kit (Geneaid Biotech Ltd, New Taipei City, Taiwan) was used, according to the manufacturer’s instructions. The extracted RNA was used to prepare cDNA using a High Capacity cDNA Reverse Transcription kit (Applied Biosystems, Thermo Fisher Scientific Inc, Carlsbad, CA, USA). The cDNA reverse-transcription reaction was performed using a programmable thermal controller (MJ Research, St. Bruno, PQ, Canada) programmed to one cycle as step1: 25°C for 10 min, step 2: 37°C for 120 min, step 3: 85°C for 5 min and step 4: 10°C after which the cDNA was stored at -20°C for future use.

#### 4.4.2 Quantification of *acrAB-tolC* gene expression by qRT-PCR

Real-time PCR was conducted to quantify the mRNA, as described by (Joshi et al., 2016). Briefly, all of the primers used in this study were designed using the National Center for Biotechnology Information (NCBI) primer BLAST software (http://www.ncbi.nlm.nih.gov/tools/primer-blast/). The generated primers were 100 to 120 bp in size and the melting temperatures of the primers were designed for 60°C, with a difference of less than 5°C for each primer pair (**Table S1**). To exclude the possibility of non-specific binding, primer sequences were analyzed by BLAST analysis (using NCBI BLAST software) against the database for the genus *Pectobacterium.* All PCR products were sequenced and found to represent the correct gene. The primer mixture for qRT-PCR contained 5 μL of Fast SYBR Green Master Mix (Applied Biosystems) and 0.8 μL (5 μM) of each forward and reverse primer. A total of 3.4 μL (17 ng) of cDNA was added to each well, so that the total reaction mixture would be 10 µL for each well. Reactions were performed using a Step One Plus Real-Time PCR system (Applied Biosystems) with the following cycling parameters: holding stage, 95°C for 20 s; cycling stage, 40 cycles of 95°C for 3 s and 60°C for 30 s; and melting curve stage, 95°C for 15 s, 60°C for 1 min and 95°C for 15 s. The data were analyzed by the comparative CT (ΔΔCT) method, with expression normalized to the expression of the reference gene *ffh*, as described by Takle et al. (2007).

## 5 Homology modeling

The model of each monomer (chain A, B, C) of *P. brasiliense* (Accession number at BLAST: WP_039274619) was constructed based on the AcrB of *E. coli* structure (PDB code: 3AOD (Nakashima et al., 2011), 80% sequence identity to *P. brasiliense*), using MODELLER (Marti-Renom et al., 2000; Webb and Sali, 2016). Sequence alignment between the target and the template was performed using the align2d python script in MODELLER v.9.20 and is presented in **Supplementary Figure 4.** After visual inspection of the alignment, models were generated using the default parameters in MODELLER (**Figure 5**). PROCHECK (Laskowski et al., 1993), was used to check the validity of the resulting models, and showed good stereochemical quality (89.0%, 86.4%, and 90.7% of residues to be in the most favorable regions of Ramachandran plots of AcrB chains A, B, and C models, respectively) and an overall G-factors of -0.14, -0.15, and -0.08 for AcrB chain A, B, and C models, respectively. The G-factor is a measure of how “normal” the protein structure is and of its stereochemical properties. A low G-factor (or very negative) indicates that the stereochemical properties correspond to a low-probability conformation of the protein. The more positive the G-factor values, the more stereochemically plausible the protein structure is. Prosa (Wiederstein and Sippl, 2007) profiles were generate and were found to be similar to those of the templates with z-score of -11.8, -11.4, and -12.09 for chain A, B, and C model, respectively. The RMSD between the models constructed in this work and the AcrB structure of *E. coli* (PDB code: 3AOD) is less than 3.0 Å.

### 5.1 Docking

Prior to docking, the protein model was process using the protein preparation wizard (Sastry et al., 2013) as implemented in Schrodinger’s Maestro to: (A) Add missing atoms/sidechains, (B) Optimize the orientation of side chains of Gln, Asn, and His, (C) Set the correct protonation states of titratable residues based on predicted pKa values. Ligands were prepared for docking using Schordinger’s LigPrep program to set correct protonation states. Docking was performed with the Glide program using the SP setting and default parameters. To validate the docking procedure, we first attempted to reproduce the crystallographic binding modes of minocycline and of rifampicin within the crystal structure of *E. coli* AcrB (PDB code 3AOD). These were satisfactorily reproduced to within 0.7 Å and 2.4 Å, respectively (based on lowest energy poses). For docking of EPIs, plant-derived compounds and plant-derived compounds in the presence of PAβN, the docking box was defined based on residues that are in radius of 4 Å from the docked minocycline in the distal pocket (chain A), and docked rifampicin in the proximal pocket (chain C) of the AcrB model.

In the docking studies we considered binding sites found in both the “access” and the “binding” states. These sites represent different intermediate states along the transport pathway for which we have crystal structure with inhibitors. Inhibition of one of these states is likely to affect the function of the efflux pump. We didn’t consider the binding site in the “extrusion” state, since the crystal structure corresponding to the state was solved in the absence of a ligand.

## 6 Infection assays

To assess the effects of each compound and EPIs on Pb1692 virulence, and to depict a potential synergistic effect of the different combinations on infection capability of Pb1692, the severity of disease symptoms development was assessed in two host plants, *Zantedeschia aethiopica* (calla lily) and *Solanum tuberosum* (potato). The infection procedure was previously described (Luzzatto et al., 2007; Yishay et al., 2008; Joshi et al., 2016). For the assays, fully expanded young leaves of calla lily, or small (25−50 g) potato tubers, were surface-sterilized by soaking in 0.5% sodium hypochlorite solution for 20 min. Then, the samples were washed twice with sterile, double-distilled H_2_O and air-dried under a laminar flow hood. Calla lily leaf discs 20 mm in diameter were excised and transferred to a Petri dish containing MS medium. Whole baby potato tubers cultivar ‘Nicola’ were used for the infection assay. Both the leaf discs and the potato tubers were pierced at the center with a sterile tip. Bacteria grown for 16 h, 28°C in liquid LB were diluted to 10^8^ CFU/mL in sterile, double-distilled H_2_O with or without the tested compound and incubated for 2 h at 28°C on a 150-rpm incubator shaker. After 2 h, leaf discs and potato tubers were inoculated with 10 μL of bacterial suspension (10^6^ CFU) in the presence or absence of the tested compounds. The inoculated plant material was incubated at 28°C for 15 h and 48 h for calla lily and potato tubers respectively. Disease severity was expressed as the percentage of decayed tissues relative to the total leaf disc area or for the potato tubers, and quantified as the percentage of rotten tissue based on weight. The experiments were repeated three times with 10 replicates for calla lily and 4 replicates for potato tubers.

## Data availability statement

The original contributions presented in the study are included in the article/**Supplementary Material**. Further inquiries can be directed to the corresponding author.

## Author contributions

MP, NK, and OG, conducted the experiments, analyzed the data and prepared the manuscript. NK and HS analyzed computational docking data, IY, and ZK, planed and coordinated laboratory experiments, IY, ZK and HS, wrote and critically revised the manuscript. All authors contributed to the article and approved the submitted version.
