# Supplementary material for: Inhibition of AcrAB-TolC enhances antimicrobial activity of phytochemicals in Pectobacterium brasiliense
Source: Front Plant Sci. 2023 May 9;14:1161702. doi: 10.3389/fpls.2023.1161702 (PMC10203483; doi:10.3389/fpls.2023.1161702)
Supplement: Supplementary file 1 [file DataSheet_1.docx]

**Supplementary Figures and Tables**

**Table S1.** List of primers used in the study

| Primer Name | | Sequences (5′ to 3′) | | Use | | Origin |
| --- | --- | --- | --- | --- | --- | --- |
| *acrA_F* | GCC AGA TTG ATT TGT GCG GT | | **Multidrug efflux pump subunit** | | This study | |
| *acrA_R* | ACC AAC TAC GTC AGC AAG CA | |  |  | This study | |
| *acrB_F* | CTA TGA AGA TCC CGC GAC CT | | **Multidrug efflux pump subunit** | | This study | |
| *acrB_R* | TTC ATT GAT CCT GGC GGT CT | |  |  | This study | |
| *tolC_F* | TGA GCG GTC TGA TAG GTG AC | | **Multidrug efflux pump subunit** | | This study | |
| *tolC_R* | GCT TCA CTG CAA TTG ACC CA | |  |  | This study | |
| *ffh _F* | TGG CAA GCC AAT TAA ATT CC | | Signal recognition particle subunit (housekeeping gene) | | Moleleki et al. (2017) | |
| *ffh _R* | TCC AGG AAG TCG GTC AAA TC | |  | | Moleleki et al. (2017) | |


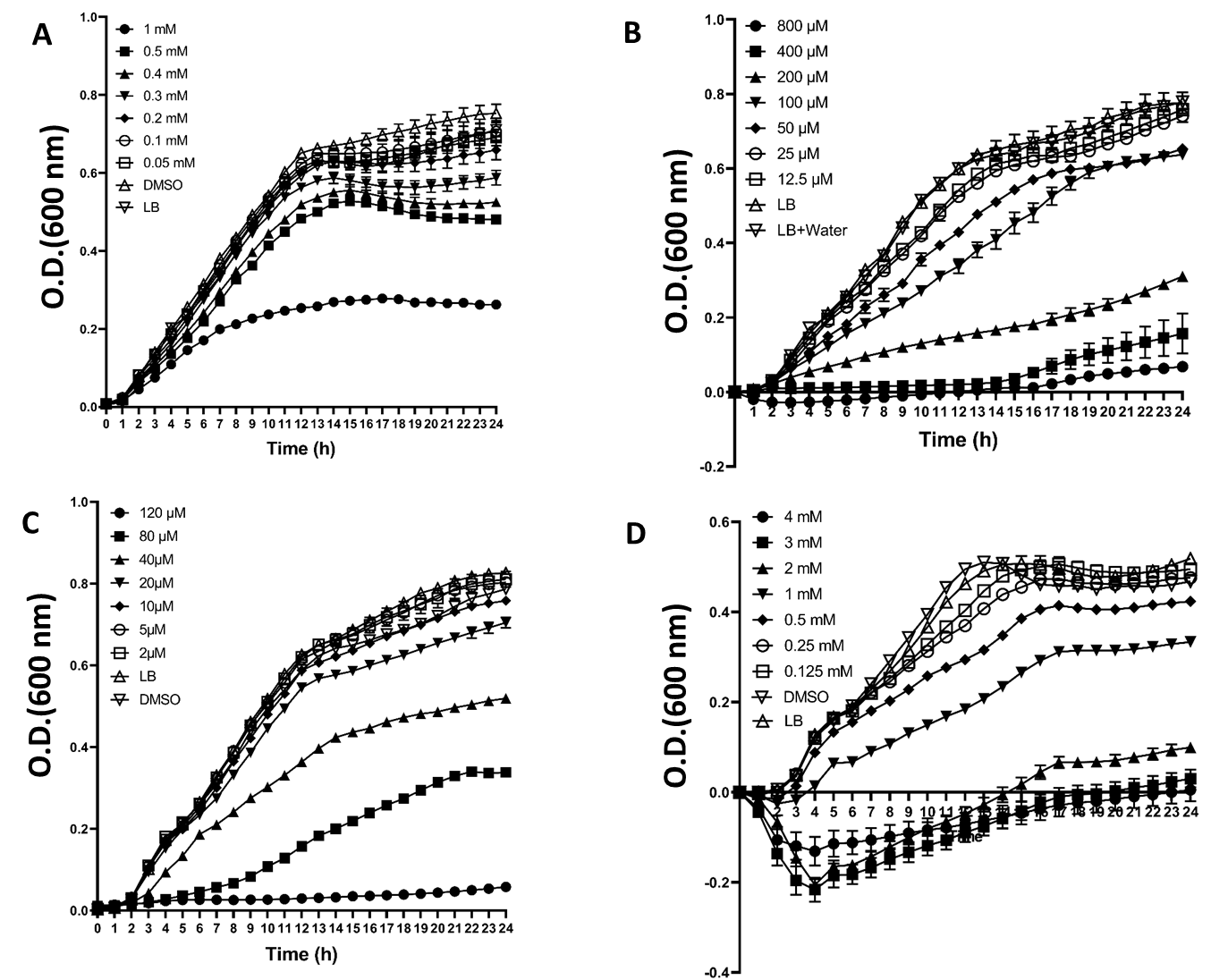


**Figure S1**. Growth curves of *Pectobacterium brasiliense* (Pb1692) in the presence of efflux pump inhibitors (EPI). Growth (OD 600 nm) was recorded in the presence of increasing concentration of A. 1-(1-naphthylmethyl)piperazine (NMP); B. Phenylalanine-arginine-β-naphthylamide (PAβN); C. Quinoline; and D. Berberine. DMSO (1%), or dH_2_O were used controls. Bacteria were grown at 28°C for 24 h and OD was recorded every hour. Each data point represents 8 replicates ± SE. Analysis was made by GraphPad Prism 8.0.


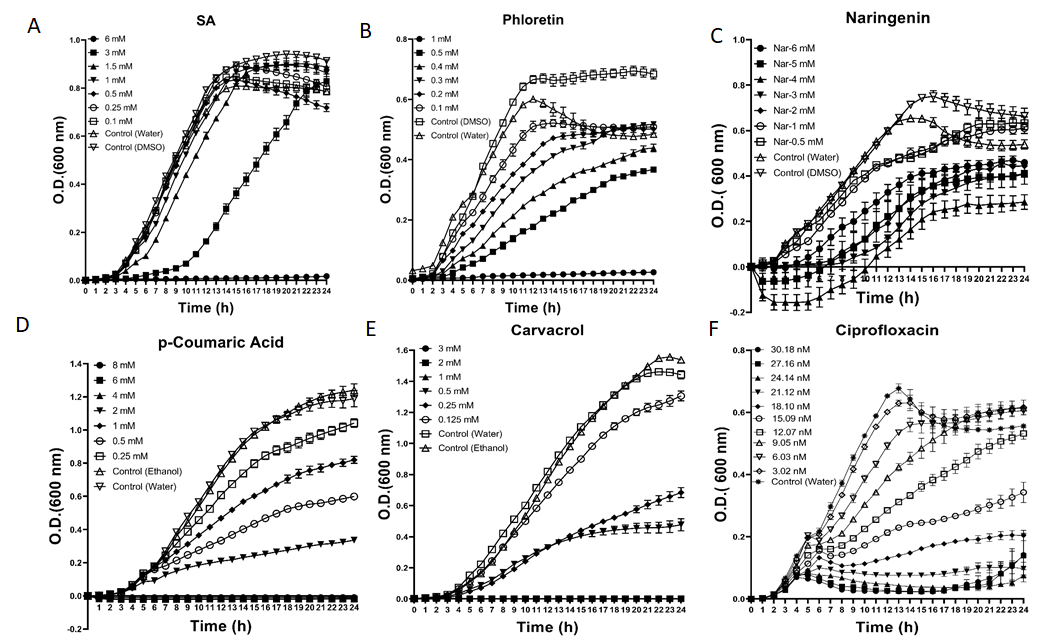


**Figure S2**. Growth curves of *Pectobacterium brasiliense* (Pb1692) in the presence of plant phenolic compounds and the antibiotic ciprofloxacin (Cip). Growth (OD 600 nm) was recorded in the presence of increasing concentration of A. Salicylic acid, B. Phloretin, C. Naringenin, D. p-Coumaric acid, E. Carvacrol, F. Ciprofloxacin. DMSO (1%) or dH_2_O were used as controls. Bacteria were grown at 28°C for 24 h and OD 600 nm was recorded every hour. Each data point represents 8 replicates ± SE. Analysis was made by GraphPad Prism 8.0.


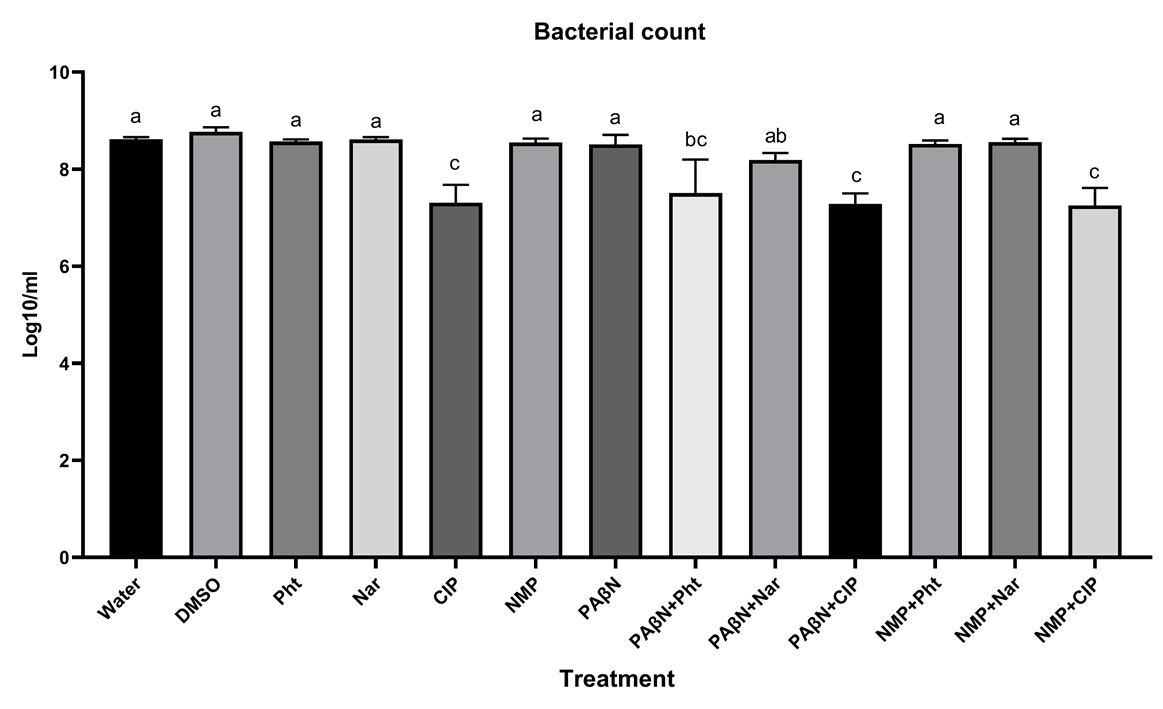


**Figure S3**. Bacterial cell counts (CFU/ml) of *Pectobacterium brasiliense* cells grown in the presence of non-lethal concentration of phloretin, naringenin and ciprofloxacin with or without the presence of efflux pump inhibitors NMP and PAβN after 2 h of incubation. DMSO (0.3%) was used as solvent and as an additional control to dH_2_O. Each data point represents the mean±standard error (SE) of 18 replicates per treatment. Treatments labeled with different letter are significantly different (p<0.05). One-way ANOVA with post hoc Tukey-Kramer HSD tests was used to analyze differences, bars not labeled with the same letter are significantly different from each other. Analysis was made by GraphPad Prism 8.0


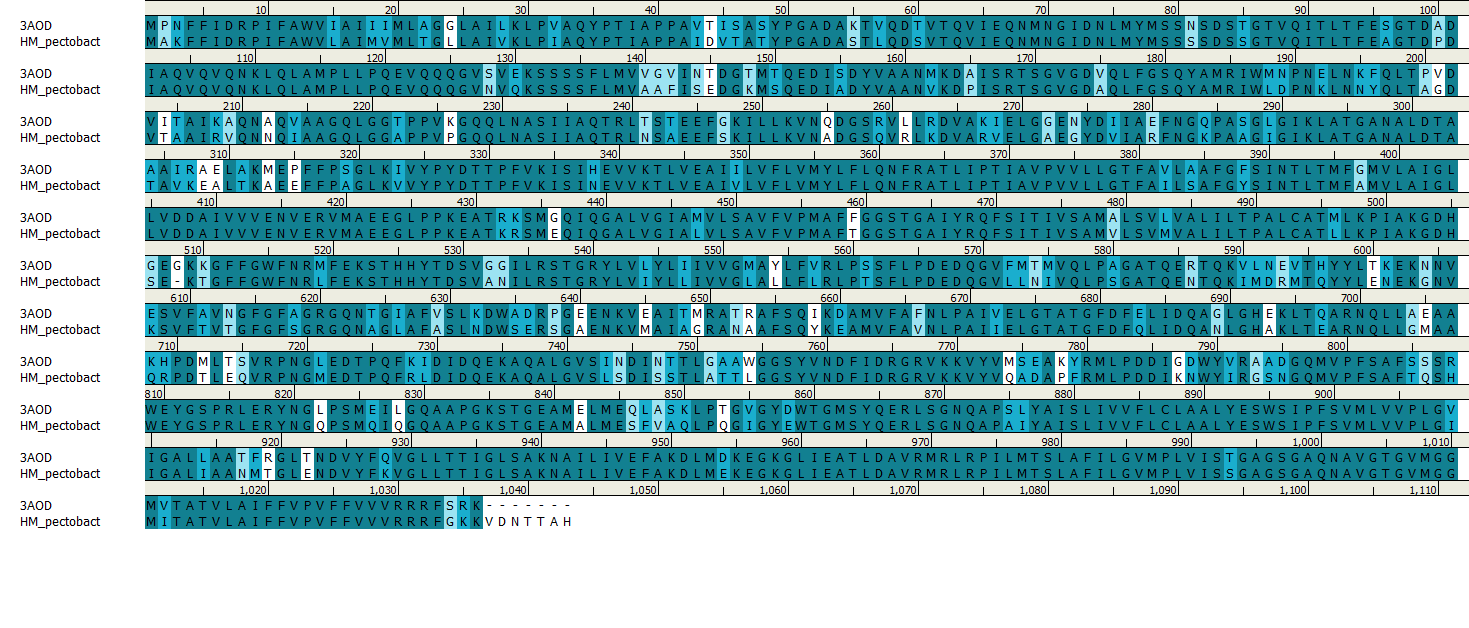


**Figure S4.** Sequence alignment of *Escherichia coli* AcrB with *Pectobacterium brasiliense* AcrB. Identical, similar and non-similar residues are colored in dark blue, light blue and white, respectively

**References**

Moleleki, L.N., Pretorius, R.G., Tanui, C.K., Mosina, G., and Theron, J. (2017). A quorum sensing-defective mutant of *Pectobacterium carotovorum* ssp. *brasiliense* 1692 is attenuated in virulence and unable to occlude xylem tissue of susceptible potato plant stems. *Mol. Plant Pathol*. 18, 32‒44.
